# Toward Precision Medicine: Gene Therapy Applications in the Management of Uveal Melanoma

**DOI:** 10.1002/cnr2.70425

**Published:** 2025-12-14

**Authors:** Alireza Azani, Vahid Ghassemifar, Zahra Mehrdad, Maryam Saberivand, Anahid Bagheripour, Safa Tahmasebi, Hossein Gharedaghi, Malihe Sharafi, Hassan Foroozand, Mohammad Saeed Soleimani Meigoli, Saba Pourali, Arash Salmaninejad, Faeze Ahmadi Beni, Qumars Behfar

**Affiliations:** ^1^ Department of Biology, Science and Research Branch Islamic Azad University Tehran Iran; ^2^ Department of Medical Genetics, School of Medicine Tehran University of Medical Sciences Tehran Iran; ^3^ Faculty of Medicine Tehran University of Medical Sciences Tehran Iran; ^4^ Student Research Committee, Department of Immunology, School of Medicine Shahid Beheshti University of Medical Sciences Tehran Iran; ^5^ School of Medicine Tehran University of Medical Science Tehran Iran; ^6^ Student Research Committee Shiraz University of Medical Sciences Shiraz Iran; ^7^ Department of Medical Genetics, School of Medical Sciences Tarbiat Modares University Tehran Iran

**Keywords:** gene therapy, non‐viral vectors, ocular melanoma, uveal melanoma, viral vectors

## Abstract

**Background:**

Uveal melanoma (UM) is the prevailing malignant tumor that develops within the eye in adults, and it has a bleak outlook because of the few treatment choices available and the high likelihood of returning after treatment. Currently, surgical intervention, radiation therapy, and a combination of both modalities are available therapeutic modalities for controlling UM. However, these techniques are associated with notable adverse effects and have limited efficacy. Therefore, there is a lack of sufficient and reliable therapies for UM, especially for advanced and metastatic UM forms. This review aims to summarize the clinical features and current therapies of UM and highlight recent progress in gene therapy approaches.

**Recent Findings:**

Significant developments in gene therapy have introduced multiple strategies for targeting UM. Gene silencing using siRNA and shRNA has shown efficacy in downregulating oncogenic pathways. CRISPR/Cas9‐based editing has enabled selective disruption of tumor‐promoting genes, sensitizing tumor cells to targeted inhibitors. Restoration of tumor‐suppressive miRNAs has reduced proliferation, migration, and invasion of UM cells in preclinical models. Suicide gene therapy has demonstrated potent cytotoxicity in xenografts. Moreover, oncolytic viruses and stem‐cell–associated delivery systems provide novel mechanisms for tumor‐selective gene expression and immune activation. Although challenges persist—such as delivery efficiency, immune responses, and genetic heterogeneity—ongoing innovations in vector design, non‐viral nanoformulations, and mutation‐guided therapies continue to enhance clinical feasibility.

**Conclusion:**

Gene therapy provides improved safety profiles and the possibility of tailored treatment strategies by integrating information on gene expression patterns and DNA alterations. In the future, gene therapy has the potential to improve the treatment of UM by targeting specific genetic mutations driving tumor growth and may offer new hope for patients with advanced stages of UM.

## 1 Introduction

Melanoma is characterized by the uncontrolled growth and proliferation of melanocytes. Among adults, ocular melanoma is the prevailing malignant neoplasm that occurs within the eye, while cutaneous melanoma is the most common variant of melanoma overall [1, 2]. Within ocular melanoma, uveal melanoma (UM) represents the most common type [3]. Each year, six to eight people per million in the Western world receive a diagnosis of UM. Fewer than 2% of patients present with metastasis, and more than 40% of patients will die as a result of disease progression [4, 5]. The severity of the disease, the condition of the eye, and the spread of metastasis to distant organs determine the treatment [6]. The liver is the most common site of metastases associated with a poor prognosis. Close to 95% of uveal melanoma patients who pass away have liver metastases [7]. Despite advances in primary melanoma detection and treatment, the fatality rate from uveal melanoma has stayed unchanged over the decades [8]. Surgery, chemotherapy, immunotherapy, and radiotherapy are currently used for the treatment of UM [9]. Enucleation is still required for eyes with large tumors or complications [3]. However, enucleation may have a detrimental rather than a positive influence on the progression of metastasis in many patients with choroid and ciliary body malignant melanoma [10]. Gene therapy is considered a novel treatment strategy for this malignancy [11]. The majority of eye cells are either dormant or slowly dividing. These are the required gene transfer targets for several eye disorders identified at the molecular level and are attractive gene therapy targets [12, 13]. Several hopeful clinical trials are being performed for many ocular diseases. Recent advances in molecular genetics have identified key driver mutations in UM, such as GNAQ, GNA11, BAP1, and CYSLTR2, highlighting opportunities for targeted interventions [14]. Gene therapy has emerged as a promising strategy capable of directly restoring or modulating gene function, inducing tumor cell death, and reshaping the tumor microenvironment through immunomodulation. Approaches such as gene replacement, silencing, suicide gene therapy, oncolytic virus‐based therapy, and stem‐cell‐mediated delivery have been explored in preclinical and early clinical studies, demonstrating feasibility and preliminary efficacy [15, 16]. These findings underscore the potential of gene therapy to complement existing treatments and serve as a second or third‐line approach, particularly for metastatic or treatment‐resistant tumors. There are multiple investigations evaluating the effectiveness of gene therapy methods for the management of UM. The present study aims to provide a comprehensive overview of the mechanisms and therapeutic potential of gene therapy in UM, and also discuss the existing challenges of gene therapy and opportunities for clinical translation and future research perspectives. “This review focuses on recent advances in gene therapy for UM, particularly studies published mainly after 2017”. Articles were selected from databases based on their relevance to the issue.

## 2 Ocular Melanoma

Noncutaneous melanomas with poor overall survival and high metastasis rates are rare but important cancers [17]. Ocular melanoma (OM) is the second most common type of melanoma, after cutaneous melanoma, and the leading common type of noncutaneous melanoma [18, 19, 20]. OM is the leading common primary intraocular malignant tumor in adults [5, 21]. OM comprises 3.7% and 73% of all melanomas and noncutaneous melanomas, respectively [18, 19]. The United States reports an age‐adjusted incidence rate of OM at 5 per 1 000 000 population [22]. OM is categorized into uveal melanoma (UM), conjunctival melanoma (CM), orbital melanoma (OM), and eyelid melanoma (EM) subtypes based on the involved anatomical regions [23]. The choroid, iris, and ciliary body give rise to UM, which accounts for the majority (83%) of OMs [24, 25]. OM is formed by melanocytes in the uveal tract or conjunctiva. OM subtypes have different genetic profiles [18, 21]. In UM, changes in the GNA11 and GNAQ genes are common. Also, the prognosis of UM depends on the mutational status of the EIF1AX, SF3B1, and BAP1 genes, which are linked to metastasis [18, 21, 26, 27, 28, 29, 30]. CM, as well as cutaneous melanoma, frequently exhibits mutations in the TERT, NRAS, and BRAF promoter genes [31]. OM mostly involves a unilateral eye [32, 33]. Mahendraraj et al. [32] found right eye involvement in 50.1% and left eye involvement in 49.8% of OM patients; moreover, they found 0.1% of OM patients with bilateral eye involvement. There are some risk factors for OM, such as welding, atypical cutaneous nevi, common cutaneous nevi, iris nevi, fair skin color, light eye color (gray or blue), cutaneous freckles, and the ability to tan. In contrast, hair color (blond or red) is not an OM risk factor [34, 35]. Early OM diagnosis leads to a better prognosis [25, 36]. Several diagnostic techniques, including enhanced depth imaging, optical coherence tomography, fluorescein angiography, fine‐needle aspiration biopsy, and ultrasonography, have been utilized in the diagnosis of OM [5, 37]. The management approach for OM depends on the condition of the involved eye, the extent of the disease, tumor biology, and the presence of metastases [5, 38]. Up to 50% of OM patients develop metastases; the median time for OM to progress to metastasis is 4.2 years, and among the different sites of metastasis, the liver is the most common initial metastatic site for OM [38, 39]. Currently, OM is treated with surgery, radiotherapy, and a combination of both [25, 32]. Surgical options in OM management include enucleation, exoresection, endoresection, and orbital exenteration [5]. There is currently no available curative or preventive treatment strategy for OM, necessitating further research on this topic [40, 41]. Studies have reported serious and major side effects following currently approved OM treatment approaches; thus, there is an unmet need to prove alternative therapeutic approaches for OM that are both more effective and safer [42, 43]. In recent years, growing evidence has shown promising results of gene therapy for OM management [42, 43, 44, 45].

## 3 Uveal Melanoma

Uveal melanoma (UM), the most prevalent adult intraocular cancer, accounts for 3%–5% of melanomas (38). Uveal melanoma, which starts from melanocytes, has different molecular causes, spreading patterns, and tumor‐immune microenvironments than cutaneous melanoma [46, 47]. Almost 90% of tumors involve the choroid, with just a small proportion affecting the ciliary body or the iris [48]. Even though both uveal and cutaneous melanoma start from neural crest‐derived cells, they have different genetic signatures and fewer mutations [49]. A study by Yavuzyigitoglu et al. [29] looked at a group of UM patients and found that 57% of the tumors had initiating hotspot mutations in GNAQ and 41% had them in GNA11. The symptoms of uveal melanoma can vary based on the tumor location and size. While some individuals may not experience any symptoms at all, others may notice changes in vision, such as blurriness or flashes of light, a black spot on the iris, or a change in the pupil's shape [50, 51]. Ophthalmologists commonly diagnose uveal melanoma via an eye examination that involves assessing both the exterior and interior of the eye, evaluating the eye's exterior for any signs of abnormal blood vessels, and using specialized equipment like binocular indirect ophthalmoscopy and slit‐lamp biomicroscopy to examine the interior [52, 53]. Whether the tumor has spread to other organs will determine the course of treatment for uveal melanoma.

## 4 Choroidal Melanoma

Choroidal melanoma is a rare and serious type of uveal melanoma which is accompanied by ciliary body and iris melanomas [54]. Certain factors, such as having light skin and eyes, being an adult male, and being of Caucasian descent, have been identified as potential risk factors for developing uveal melanoma [55, 56]. The pathogenesis of choroidal melanoma is currently unknown. However, significant progress has been made in the molecular mechanisms. Aggressive uveal melanoma has long been associated with monosomy 3 [54, 57]. Recent research has identified specific abnormalities in loci associated with high‐risk melanoma, such as chromosome 3 and 1p losses and 8q gains [58]. Current research has demonstrated the high conservation of numerous genetic mutations in the clonal proliferation of uveal melanocytes [54, 57]. For extended periods, choroidal melanomas can remain asymptomatic, leading to their accidental discovery during ophthalmoscopy. Blurred vision, photopsia, eye floaters, loss of visual field area, detectable tumors, pain, and metamorphopsia are all symptoms of choroidal melanoma. Small choroidal melanomas are often a nodular, dome‐shaped, well‐circumscribed mass underneath the retinal pigment epithelium. Choroidal melanomas may adopt increasingly irregular forms as they grow (e.g., bilobular, multilobular, or mushroom morphologies). Diffuse choroidal melanoma, characterized by lateral proliferation across the choroid with minimal elevation, is more challenging to detect and often leads to severe exudative retinal detachment [59]. There are various current treatments available for choroidal melanoma. Besides enucleation and brachytherapy, other ways to treat choroidal melanoma are through immunotherapy, cytotoxic chemotherapy, liver‐directed therapies, molecularly targeted therapies, and epigenetic modifiers. Laser therapy has also been employed as a comparatively nonsurgical treatment option for smaller and more visible malignant melanoma of the choroid, saving the eye, life, and eyesight [60]. However, gene therapy is a fast‐expanding discipline, leading to recent advances in the treatment of ocular melanoma. One such treatment is AU‐011, which is now in Phase 2 of human studies to treat choroidal melanoma using intravitreal or suprachoroidal injections followed by laser photoactivation in an office setting. Another intriguing method is to exploit GQ/11 as a new gene‐product‐specific therapeutic target for uveal melanoma therapy [61]. However, gene therapy has tremendous potential in the treatment of choroidal melanoma, and recent advances in gene editing, gene silencing, and viral vectors offer new options for creating successful and tailored gene therapy techniques. It is critical to identify mutations and possible targets for gene therapy, and current research efforts should concentrate on identifying biomarkers and developing gene therapy systems capable of efficiently targeting and eliminating choroidal melanoma cells.

## 5 Iris Melanoma

Iris melanoma is the most prevalent iris malignancy and one of the rarest types of uveal melanoma, accounting for about 2%–5% of uveal melanomas and having a better prognosis in comparison to other types of uveal melanoma [62, 63]. The prevalence of iris melanoma is approximately equal in men and women, with an incidence of 1–9 cases per million people, and it mostly occurs in middle‐aged and lighter‐eyed individuals. Most iris melanomas are spindle cell types, while epithelioid cells and mixed types have a higher risk of metastasis [64, 65]. During the early stages of the disease and when the tumor is small, iris melanoma may remain asymptomatic. However, it can also cause symptoms such as ectropion of the uvea, glaucoma, papillary distortion, tumor vascularization, sector cataract, chronic uveitis, irreversible optic nerve damage, and hyphema [63]. We choose the treatment type with a preference for preserving vision. The main treatments for iris melanoma are local surgical resection, enucleation, plaque brachytherapy, and proton beam therapy.

## 6 Ciliary Body Melanoma

Melanoma of the ciliary body, a rarely but aggressively observed kind of OM, is reported in 1 of 10 cases of all intraocular melanomas with a severely poor prognosis. The continued contractions of the ciliary muscle and its being rich in vascularization in this area lead to a faster hematological metastasis of ciliary body melanoma. Another problem of this malignancy is late diagnosis, which is rooted in a lack of clinical signs in the early stages that makes it impossible to detect the tumor within an eye examination [66, 67]. However, patients with ciliary body melanoma may first experience symptoms such as reduced visual acuity; it may also be accompanied by a visible mass when the tumor extends into the anterior segment [68]. The management of ciliary body melanoma depends on several factors, including tumor size and location, tumor invasion, presence of extraocular extension, the degree of visual impairment, etc. Plaque brachytherapy is applied for localized tumors, and proton beam radiotherapy is used for larger or more posterior tumors [69, 70]. Surgical resection in the required condition and enucleation when eye preservation is not feasible are also considered, as Lai et al. presented a young, healthy Chinese woman who experienced a one‐week history of reduced vision in her left eye [66]. Although research into gene therapy for ocular melanomas in general is ongoing, there is no specific investigation exploring gene therapy in the treatment of ciliary body melanoma.

## 7 Current Therapies for UM

**FIGURE 1:**
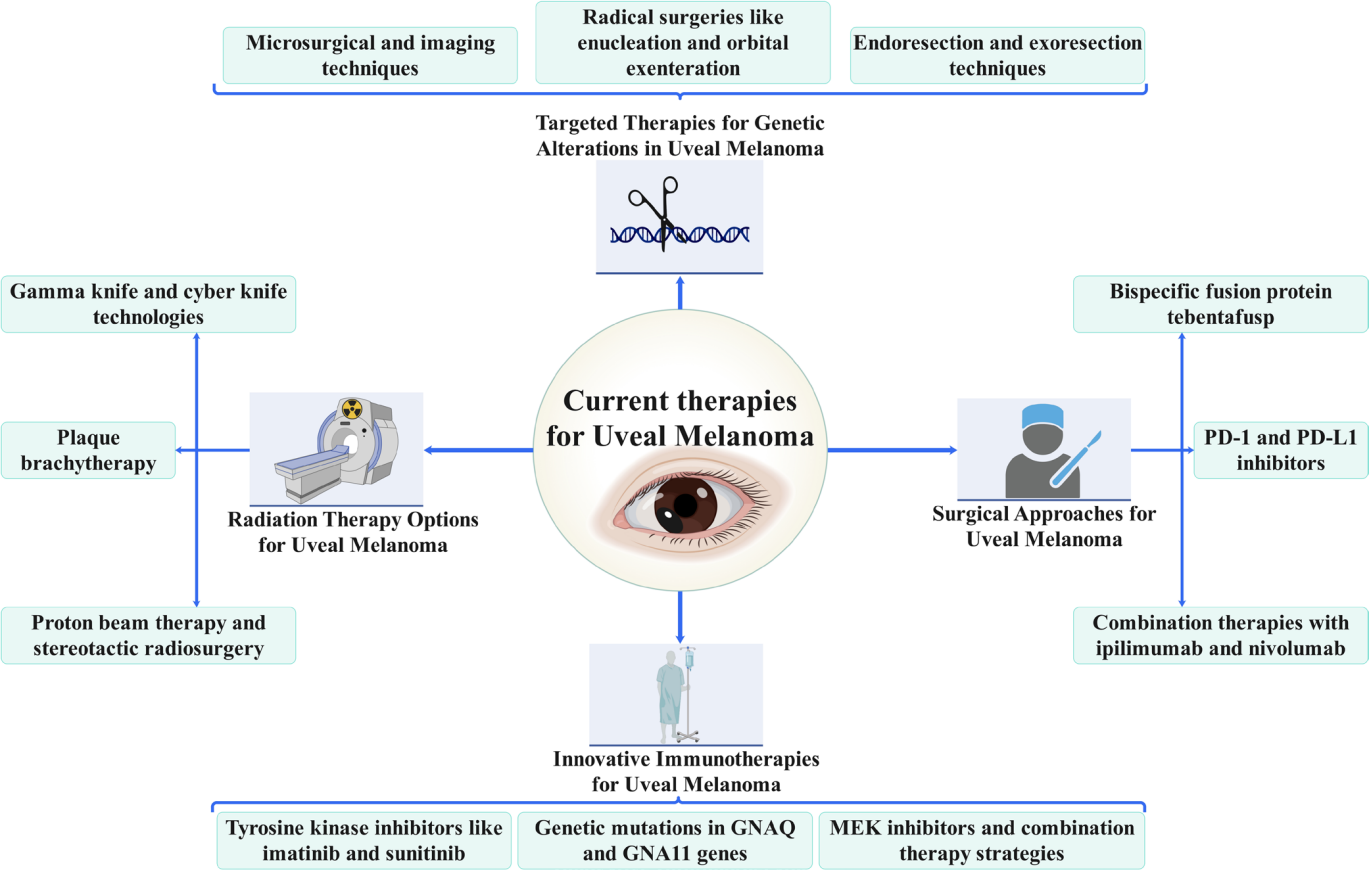
Overview of current therapeutic strategies for uveal melanoma. Standard treatment options include surgical excision, radiotherapy (e.g., plaque brachytherapy, proton beam therapy), and transpupillary thermotherapy. In recent years, targeted therapies and immunotherapy have emerged as potential options, particularly for metastatic or treatment‐resistant cases.

The treatment options for UM include globally conserving surgeries, such as endoresection or exoresection, as well as more radical procedures like ocular enucleation or orbital exenteration (Figure 1). Scleral flap exoresection and vitreous cutter endoresection are two specific surgical techniques used to remove choroidal melanomas [71, 72]. This treatment may be able to eradicate the tumor, preserve the globe, and potentially restore usable vision. UMs that are not suitable for radiotherapy due to their location or size may undergo local excision [71]. It also provides tumor samples for histological and cytogenetic testing [73, 74]. Prior to plaque brachytherapy, enucleation was the first‐line treatment for UM. People commonly use enucleation to treat advanced tumors that radiation cannot effectively treat. Radiation is another ocular melanoma treatment that different types of radiation can kill or stop melanoma growth [75, 76]. Because the eye is tiny and melanomas are radioresistant, oncologists treating uveal melanoma must provide high doses in a targeted area. Proton beam therapy and stereotactic radiosurgery are two treatments that use external beams [77]. However, radiotherapy can cause radiation‐induced retinopathy or visual neuropathy, and also painful secondary glaucoma, blurred vision, or orbital invasion [76, 78]. Plaque brachytherapy is another technique for surgically administering radiation to the eye. To treat uveal melanoma, all approaches have proven to be effective. However, obstacles to achieving proper dose distribution and managing long‐term negative effects exist [79].

Targeted therapy in UM is an evolving field, with recent advancements focusing on personalized approaches based on specific genetic mutations [80]. C‐kit is expressed in most patients suffering from metastatic UM. In a clinical trial led by Mahipal, the toxicity and efficacy of sunitinib malate, a multitarget tyrosine kinase inhibitor, were examined in 20 patients with metastatic UM who express c‐kit. Their data supported the potential of sunitinib in improving clinical outcomes of patients [81]. UM often has changes in the GNAQ and GNA11 genes, which may turn on mitogen‐activated protein kinase (MAPK) like BRAF mutations do in cutaneous melanoma [28, 82]. Selumetinib, designed to inhibit MEK, is used as a monotherapy or in combination with chemotherapy to treat metastatic UV [83, 84]. However, monotherapy commonly causes resistance, encouraging medication sequencing and combination regimens. However, the combination prolonged median non‐progression survival. Genetic profile stratification can also optimize treatment outcomes by personalizing therapy [85].

Immunotherapy is also successful in the treatment of UM [21]. CAR‐T cell treatment, a novel iteration of cancer immunotherapy, involves manipulating a patient's T cells to enhance their ability to identify and eradicate malignant cells. The procedure entails the introduction of retroviral vectors, such as lentivirus, into enriched T cells. These vectors carry the required genetic material or facilitate gene editing techniques to insert the desired gene change. The ultimate goal is to induce the expression of chimeric antigen receptors (CARs) on T cells. Genetically modified T‐cells subsequently proliferate to a level suitable for therapeutic application [86]. CAR T‐cells, when combined with intact surface antigens, can recognize antigens without being limited by the context of HLA molecules. As a result, they are considered to be highly suitable for patient populations with diverse HLA profiles [87, 88]. A study by Forsberg et al. [89] confirmed that HER2 mRNA was the only molecule expressed at a significant level in most cases of UM, especially when it came to well‐known chimeric antigen receptor T‐cell (CAR‐T) targets. Furthermore, the study demonstrated that UM exhibited a target‐specific response to HER2 CAR‐T cells. One of the main goals of vaccinations is to specifically activate CD4+ T‐cells, which are very important for both CD8+ T‐cell‐mediated protective immunity and the development of immunological memory [90, 91, 92]. The discovery and characterization of signaling pathways, as well as the connections between the tumor and the immunological microenvironment, have significantly changed the therapeutic approaches for melanoma [93, 94]. Finding the RAS–RAF–MEK–ERK (MAP kinase) signaling pathway and then targeting it has been a big step forward in treating advanced melanoma, especially when it comes to completely resected advanced‐stage melanoma therapy [95, 96]. The current therapeutic strategies for metastatic cancer, namely immunotherapy and BRAF+MEK inhibitors, have demonstrated a maximum 5‐year survival rate of 60%. However, a significant proportion of patients relapse during treatment as a result of acquired resistance mechanisms [97]. As a result, understanding the biology of the BRAF gene is crucial for identifying the primary and secondary causes of acquired resistance, as well as the molecular pathways currently under investigation in preclinical and clinical trials [97]. All clinical trials related to ocular melanomas and uveal melanomas are summarized in Tables 1 and 2.

**TABLE 1:** Recruiting, completed, and terminated clinical trials of ocular melanoma.

Study title	Intervention	Condition	Study type	Phase	References
STA‐9090 (ganetespib) in metastatic ocular melanoma	STA‐9090	Ocular melanoma	Interventional/single group assignment non‐randomized	Phase 2	NCT01200238, https://clinicaltrials.gov/study/NCT01200238 [98]
Immunotherapy using tumor infiltrating lymphocytes for patients with metastatic ocular melanoma	Aldesleukin/cyclophosphamide/fludarabine/young tumor infiltrating lymphocytes (TIL)	Metastatic ocular melanoma Metastatic uveal melanoma	Interventional/parallel assignment non‐randomized	Phase 2	NCT01814046, https://clinicaltrials.gov/study/NCT01814046 [99]
Cabozantinib‐S‐malate compared with temozolomide or dacarbazine in treating patients with metastatic melanoma of the eye that cannot be removed by surgery	Cabozantinib S‐malate/dacarbazine/temozolomide Other: Laboratory biomarker analysis	Recurrent uveal melanoma Stage III, IIIA, IIIB, IIIC, IV uveal melanoma AJCC v7	Interventional/parallel assignment randomized	Phase 2	NCT01835145, https://clinicaltrials.gov/study/NCT01835145 [100]
Radiation and combination immunotherapy for melanoma	Aldesleukin: All patients/nivolumab: Cohort 1 (cutaneous)/nivolumab: Cohort 2 (ocular)/ipilimumab: Cohort 2 (ocular)	Metastatic melanoma	Interventional/parallel assignment non‐randomized	Phase 2	NCT03850691, https://clinicaltrials.gov/study/NCT03850691 [101]
Paclitaxel albumin‐stabilized nanoparticle formulation in treating patients with metastatic melanoma of the eye that cannot be removed by surgery	Nab‐paclitaxel	Intraocular melanoma	Interventional/single group assignment	Phase 2	NCT00738361, https://www.clinicaltrials.gov/study/NCT00738361 [102]
Neoadjuvant and adjuvant checkpoint blockade	Ipilimumab/nivolumab/relatlimab Other: Laboratory biomarker analysis Procedure: Therapeutic conventional surgery	Cutaneous melanoma Mucosal melanoma Ocular melanoma Stage III acral lentiginous melanoma AJCC v7 Stage IIIB cutaneous melanoma AJCC v7 Stage IIIB uveal melanoma AJCC v7 Stage IIIC cutaneous melanoma AJCC v7 Stage IIIC uveal melanoma AJCC v7 Stage IV Acral lentiginous melanoma AJCC v6 and v7 Stage IV cutaneous melanoma AJCC v6 and v7 Stage IV uveal melanoma AJCC v7	Interventional/parallel assignment randomized	Phase 2	NCT02519322, https://clinicaltrials.gov/study/NCT02519322 [103]
A Vaccine (CDX‐1401) with or without a biologic drug (CDX‐301) for the treatment of patients with Stage IIB‐IV melanoma	DEC‐205/NY‐ESO‐1 fusion protein CDX‐1401/poly ICLC/recombinant Flt3 Ligand	Cutaneous melanoma Melanoma Melanoma of unknown primary Mucosal melanoma Ocular melanoma Stage IIB cutaneous melanoma AJCC v6 and v7 Stage IIC cutaneous melanoma AJCC v6 and v7 Stage III cutaneous melanoma AJCC v7 Stage IIIA cutaneous melanoma AJCC v7 Stage IIIB cutaneous melanoma AJCC v7 Stage IIIC cutaneous melanoma AJCC v7 Stage IV cutaneous melanoma AJCC v6 and v7	Interventional/parallel assignment randomized	Phase 2	NCT02129075, https://clinicaltrials.gov/study/NCT02129075 [104]
Sargramostim, vaccine therapy, or sargramostim and vaccine therapy in preventing disease recurrence in patients with melanoma that has been removed by surgery	Sargramostim/tyrosinase peptide/placebo Other: Laboratory biomarker analysis	Iris melanoma Medium/large size posterior uveal melanoma Mucosal melanoma Ocular melanoma with extraocular extension Recurrent melanoma Recurrent uveal melanoma Small size posterior uveal melanoma Stage IIA cutaneous melanoma AJCC v6 and v7 Stage IIA uveal melanoma AJCC v7 Stage IIB cutaneous melanoma AJCC v6 and v7 Stage IIB UVEAL MELANOMA AJCC v7 Stage IIC cutaneous melanoma AJCC v6 and v7 Stage IIIA cutaneous melanoma AJCC v7 Stage IIIA uveal melanoma AJCC v7 Stage IIIB cutaneous melanoma AJCC v7 Stage IIIB uveal melanoma AJCC v7 Stage IIIC cutaneous melanoma AJCC v7 Stage IIIC uveal melanoma AJCC v7 Stage IV cutaneous melanoma AJCC v6 and v7 Stage IV uveal melanoma AJCC v7	Interventional/factorial assignment randomized	Phase 3	NCT01989572, https://clinicaltrials.gov/study/NCT01989572 [105]
A Phase Ib/II study of AEB071 and MEK162 in adult patients with metastatic uveal melanoma	AEB071/MEK162	Uveal melanoma	Interventional/parallel assignment randomized	Phase 1 Phase 2	NCT01801358, https://clinicaltrials.gov/study/NCT01801358 [106]
Study of OX40 agonist PF‐04518600 alone and in combination with 4‐1BB agonist PF‐05082566	PF‐04518600/PF‐04518600 plus PF‐05082566	Neoplasms	Interventional/single group assignment non‐randomized	Phase 1	NCT02315066, https://clinicaltrials.gov/study/NCT02315066 [107]

**TABLE 2:** Recruiting, completed, and terminated Clinical trials of Uveal Melanoma.

Study title	Intervention	Condition	Study type	Phase	References
Study of PAC‐1 and entrectinib for patients with metastatic uveal melanoma	PAC‐1/entrectinib	Uveal melanoma	Interventional/single group assignment	Phase 1 Phase 2	NCT04589832, https://clinicaltrials.gov/study/NCT04589832 [108]
Treatment with intravitreal avastin for large uveal melanomas	AVASTIN	Uveal melanoma	Interventional/single group assignment	Not Applicable (NA)	NCT00596362, https://clinicaltrials.gov/study/NCT00596362 [109]
A Phase Ib/II Study of AEB071 and MEK162 in adult patients with metastatic uveal melanoma	AEB071/MEK162	Uveal melanoma	Interventional/parallel assignment randomized	Phase 1 Phase 2	NCT01801358, https://clinicaltrials.gov/study/NCT01801358 [106]
Crizotinib in high‐risk uveal melanoma following definitive therapy	Crizotinib	Uveal melanoma	Interventional/single group assignment	Phase 2	NCT02223819, https://clinicaltrials.gov/study/NCT02223819 [110]
A Study of the intra‐patient escalation dosing regimen with IMCgp100 in patients with advanced uveal melanoma	IMCgp100	Uveal melanoma	Interventional/single group assignment non‐randomized	Phase 1 Phase 2	NCT02570308, https://clinicaltrials.gov/study/NCT02570308 [111]
RAD001 (everolimus) and pasireotide (SOM230) LAR in patients with advanced uveal melanoma	RAD001 (everolimus)/pasireotide (SOM230) LAR	Uveal melanoma	Interventional/single group assignment	Phase 2	NCT01252251, https://clinicaltrials.gov/study/NCT01252251 [112]
Selumetinib (AZD6244: ARRY‐142886) (Hyd‐Sulfate) in metastatic uveal melanoma (SUMIT)	75 mg selumetinib/dacarbazine/placebo	Metastatic Uveal melanoma	Interventional/parallel assignment randomized	Phase 3	NCT01974752, https://clinicaltrials.gov/study/NCT01974752 [113]
Glembatumumab vedotin in treating patients with metastatic or locally recurrent uveal melanoma	Glembatumumab vedotin Other: Laboratory biomarker analysis other: pharmacological study	Recurrent uveal melanoma Stage IV uveal melanoma AJCC v7	Interventional/single group assignment	Phase 2	NCT02363283, https://clinicaltrials.gov/study/NCT02363283 [114]
Trametinib with or without GSK2141795 in treating patients with metastatic uveal melanoma	Trametinib/uprosertib Other: Laboratory biomarker analysis Other: Pharmacological study	Recurrent Uveal Melanoma Stage IV Uveal Melanoma AJCC v7	Interventional/parallel assignment randomized	Phase 2	NCT01979523, https://clinicaltrials.gov/study/NCT01979523 [115]
Nivolumab and ipilimumab in treating patients with metastatic uveal melanoma	Ipilimumab/nivolumab Other: Laboratory biomarker analysis	Metastatic Uveal Melanoma Stage IV Uveal Melanoma AJCC v7	Interventional/single group assignment	Phase 2	NCT01585194, https://clinicaltrials.gov/study/NCT01585194 [116]
Safety and Efficacy of Marqibo in Metastatic Malignant Uveal Melanoma	Marqibo (vincristine sulfate liposomes injection)	Metastatic malignant uveal melanoma	Interventional/sequential assignment non‐randomized	Phase 2	NCT00506142, https://clinicaltrials.gov/study/NCT00506142 [117]
Study evaluating single and repeated intravitreal doses of ICON‐1 in patients with uveal melanoma	ICON‐1	Uveal melanoma choroid neoplasm	Interventional/parallel assignment non‐randomized	Phase 1	NCT02771340, https://clinicaltrials.gov/study/NCT02771340 [118]
Safety & activity of Controllable PRAME‐TCR therapy in previously treated AML/MDS or metastatic uveal melanoma	BPX‐701/rimiducid	Acute myeloid leukemia Myelodysplastic syndrome Uveal melanoma	Interventional/single group assignment non‐randomized	Phase 1 Phase 2	NCT02743611, https://clinicaltrials.gov/study/NCT02743611 [119]
Pembrolizumab in treating patients with advanced uveal melanoma	Pembrolizumab Other: Laboratory biomarker analysis	Uveal melanoma	Interventional/single group assignment	Phase 2	NCT02359851, https://www.clinicaltrials.gov/study/NCT02359851 [120]
Dexamethasone intravitreal implant for treatment of macular edema after plaque radiotherapy of uveal melanoma	Ozurdex/bevacizumab	Macular edema Cystoid macular edema Uveal melanoma Radiation maculopathy Radiation retinopathy	Interventional/single group assignment randomized	Phase 2	NCT01471054, https://clinicaltrials.gov/study/NCT01471054 [121]
Radiation therapy in preventing liver metastases in patients with uveal melanoma who have monosomy 3 or decision Dx Class 2 disease and are more likely to develop liver metastases	Radiation: External beam radiation therapy Other: Laboratory biomarker analysis	Iris melanoma uveal melanoma	Interventional/single group assignment	Not applicable	NCT02336763, https://www.clinicaltrials.gov/study/NCT02336763 [122]
Dacarbazine and recombinant interferon Alfa‐2b in treating patients with primary uveal melanoma with genetic imbalance	Recombinant interferon alfa‐2b/dacarbazine Other: Laboratory biomarker analysis	Ciliary body and choroid melanoma, medium/large size Ciliary body and choroid melanoma, small size Iris melanoma Recurrent intraocular melanoma	Interventional/single group assignment	Phase 2	NCT01100528, https://clinicaltrials.gov/study/NCT01100528 [123]
Genasense, carboplatin, paclitaxel (GCP) combination in uveal melanoma	Genasense/paclitaxel/carboplatin	Melanoma	Interventional/single group assignment	Phase 2	NCT01200342, https://clinicaltrials.gov/study/NCT01200342 [124]
Immunotherapy using tumor infiltrating lymphocytes for patients with metastatic ocular melanoma	Aldesleukin/cyclophosphamide/fludarabine/young tumor infiltrating lymphocytes (TIL)	Metastatic ocular melanoma Metastatic uveal melanoma	Interventional/parallel assignment non‐randomized	Phase 2	NCT01814046, https://clinicaltrials.gov/study/NCT01814046 [99]
Temozolomide or selumetinib in treating patients with metastatic melanoma of the eye	Dacarbazine/selumetinib/temozolomide Other: Laboratory biomarker analysis Other: quality‐of‐life assessment	Iris melanoma Medium/large size posterior uveal melanoma Ocular melanoma with extraocular extension Recurrent uveal melanoma Small size posterior uveal melanoma Stage IV uveal melanoma	Interventional/parallel assignment randomized	Phase 2	NCT01143402, https://clinicaltrials.gov/study/NCT01143402 [125]
Sorafenib, carboplatin, and paclitaxel in treating patients with stage IV melanoma of the eye	Carboplatin/paclitaxel/sorafenib tosylate	Ciliary body and choroid melanoma, medium/large size Extraocular extension melanoma Iris melanoma Metastatic intraocular melanoma Recurrent intraocular melanoma	Interventional/single group assignment	Phase 2	NCT00329641, https://www.clinicaltrials.gov/study/NCT00329641 [126]
Cixutumumab in treating patients with metastatic melanoma of the eye	Cixutumumab Other: Laboratory biomarker analysis	Ciliary body and choroid melanoma, medium/large size Ciliary body and choroid melanoma, small size Iris melanoma Metastatic intraocular melanoma Recurrent intraocular melanoma Stage IV Intraocular Melanoma	Interventional/single group assignment	Phase 2	NCT01413191, https://www.clinicaltrials.gov/study/NCT01413191 [127]
Vaccine therapy in treating patients with Stage IIB, Stage IIC, Stage III, or Stage IV melanoma	Mouse gp100 plasmid DNA vaccine Device: The dermal PowderMed devices Other: intramuscularly (IM injection)	Intraocular melanoma Melanoma (Skin)	Interventional/parallel assignment randomized	Phase 1	NCT00398073, https://www.clinicaltrials.gov/study/NCT00398073 [128]
Monoclonal antibody therapy and vaccine therapy in treating patients with resected Stage III or Stage IV melanoma	Ipilimumab/tyrosinase/gp100/MART‐1 Peptides	Intraocular melanoma Melanoma (Skin)	Interventional/single group assignment	Phase 2	NCT00084656, https://clinicaltrials.gov/study/NCT00084656 [129]
A trial of niraparib in BAP1 and other DNA damage response (DDR) deficient neoplasms (UF‐STO‐ETI‐001)	Niraparib	Mesothelioma uveal melanoma renal cell carcinoma cholangiocarcinoma	Interventional/parallel assignment non‐randomized	Phase 2	NCT03207347, https://clinicaltrials.gov/study/NCT03207347 [130]
Cabozantinib‐S‐Malate compared with temozolomide or dacarbazine in treating patients with metastatic melanoma of the eye that cannot be removed by surgery	Cabozantinib S‐malate/dacarbazine/temozolomide Other: Laboratory biomarker analysis	recurrent uveal melanoma Stage III uveal melanoma AJCC v7 Stage IIIA uveal melanoma AJCC v7 Stage IIIB Uveal Melanoma AJCC v7 Stage IIIC Uveal Melanoma AJCC v7 Stage IV uveal melanoma AJCC v7	Interventional/parallel assignment randomized	Phase 2	NCT01835145, https://clinicaltrials.gov/study/NCT01835145 [131]
Sargramostim, vaccine therapy, or sargramostim and vaccine therapy in preventing disease recurrence in patients with melanoma that has been removed by surgery	Sargramostim/tyrosinase peptide/placebo Other: Laboratory biomarker analysis	Iris melanoma Medium/large size posterior uveal melanoma Mucosal melanoma Ocular melanoma with extraocular extension Recurrent melanoma Recurrent uveal melanoma Small size posterior uveal melanoma Stage IIA cutaneous Melanoma AJCC v6 and v7 Stage IIA uveal melanoma AJCC v7 Stage IIB cutaneous melanoma AJCC v6 and v7 Stage IIB uveal melanoma AJCC v7 Stage IIC cutaneous melanoma AJCC v6 and v7 Stage IIIA cutaneous melanoma AJCC v7 Stage IIIA uveal melanoma AJCC v7 Stage IIIB cutaneous melanoma AJCC v7 Stage IIIB uveal melanoma AJCC v7 Stage IIIC cutaneous melanoma AJCC v7 Stage IIIC uveal melanoma AJCC v7 Stage IV cutaneous melanoma AJCC v6 and v7 Stage IV uveal melanoma AJCC v7	Interventional/factorial assignment randomized	Phase 3	NCT01989572, https://clinicaltrials.gov/study/NCT01989572 [105]
Nab‐paclitaxel and bevacizumab or ipilimumab as first‐line therapy in treating patients with Stage IV melanoma that cannot be removed by surgery	Bevacizumab/ipilimumab/nab‐paclitaxel Other: Laboratory biomarker analysis Other: Pharmacological study	Metastatic melanoma Mucosal melanoma Stage IV cutaneous melanoma AJCC v6 and v7 Stage IV uveal melanoma AJCC v7 Unresectable melanoma	Interventional/crossover assignment randomized	Phase 2	NCT02158520, https://clinicaltrials.gov/study/NCT02158520 [132]
Vorinostat in treating patients with metastatic or unresectable melanoma	Vorinostat	Ciliary body and choroid melanoma, medium/large size Extraocular extension melanoma Iris melanoma Uveal melanoma Recurrent intraocular melanoma Recurrent melanoma Stage IV melanoma	Interventional/single group assignment	Phase 2	NCT00121225, https://clinicaltrials.gov/study/NCT00121225 [133]
VEGF trap in treating patients with recurrent Stage III or Stage IV melanoma that cannot be removed by surgery	ziv‐aflibercept Other: pharmacological study	Ciliary body and choroid melanoma, medium/large size Extraocular extension melanoma Iris melanoma Metastatic intraocular melanoma Recurrent intraocular melanoma Recurrent melanoma Stage III melanoma Stage IV melanoma	Interventional/single group assignment	Phase 2	NCT00450255, https://clinicaltrials.gov/study/NCT00450255 [134]
Iodine I 131 monoclonal antibody 3F8 in treating patients with central nervous system cancer or leptomeningeal cancer	Genetic: DNA analysis Other: immunologic technique Other: pharmacological study Radiation: iodine I 131 monoclonal antibody 3F8 Radiation: 131I‐3F8	Brain and central nervous system tumors Intraocular melanoma Lung cancer Melanoma (Skin) Metastatic cancer Neuroblastoma Ovarian cancer Retinoblastoma Sarcoma Small Intestine Cancer	Interventional/single group assignment	Phase 2	NCT00445965, https://www.clinicaltrials.gov/study/NCT00445965 [135]
AZD2171 in treating patients with recurrent or Stage IV melanoma	Cediranib maleate	Acral lentiginous malignant melanoma Ciliary body and choroid melanoma, medium/large size Ciliary body and choroid melanoma, small size Extraocular extension melanoma Intraocular melanoma Iris melanoma Lentigo maligna malignant melanoma Recurrent melanoma Stage, intraocular melanoma Stage IV melanoma Superficial spreading malignant melanoma	Interventional/single group assignment	Phase 2	NCT00243061, https://clinicaltrials.gov/study/NCT00243061 [136]
Melanoma vaccine with peptides and leuprolide	Leuprolide/GP100: 209–217 (210 M) peptide/MAGE‐3 Peptide	Melanoma	Interventional/parallel assignment randomized	Phase 2	NCT00254397, https://clinicaltrials.gov/study/NCT00254397 [137]

To conclude, traditional methods for the treatment of UM have played a role in the improvement of the patient's situation. However, these methods have limitations. Surgery is the first step to treat primary tumors, but it is not efficient once the tumor becomes metastatic. Chemotherapy is usually accompanied by resistance and side effects in UM patients. Immunotherapies, such as checkpoint inhibitors (e.g., anti‐CTLA‐4 and anti‐PD‐1 antibodies), have significantly improved outcomes; however, the efficiency of immunotherapy in UM is poorer than its efficiency in skin melanoma, and a substantial subset of patients develops resistance over time, and low response rates are observed in clinical trials [138, 139, 140]. Targeted therapy using BRAF and MEK inhibitors is effective in patients with specific mutations, but often leads to resistance over time [141]. A Phase 1 dose‐escalation trial evaluating the MEK inhibitor TAK‐733 in 51 patients with advanced solid tumors, including 12 with uveal melanoma, demonstrated limited anti‐cancer effects [142]. These challenges highlight the necessity of a new approach to melanoma treatment that effectively overcomes these issues.

## 8 Gene Therapy

Gene therapy involves adding functional equivalents to help, repair, or modify genes that are not functioning properly. The goal is to treat conditions where genes are not working at all or are working at low levels [143, 144]. The gene therapy process can be performed through gene silencing using microRNA (miRNA), small interfering RNA (siRNA), and short hairpin RNA (shRNA). This approach utilizes the natural cellular mechanism of RNA interference to degrade mRNA transcripts associated with mutant oncogenes such as GNAQ, thereby reducing their expression. Recently, McCall et al. designed an allele‐specific siRNA targeting the GNAQ gene with the Q209L mutation, which was transfected to UM cell lines, as well as an AAV carrying a shRNA targeting this allele. Their finding indicated a significant reduction in clonogenic survival, mediated by downregulation of YAP activity in a UM cell line with a mutation in Q209L in the GNAQ gene, after employing siRNA or shRNA [145]. Also, DERL1, a membrane protein that identifies substrates in the endoplasmic reticulum (ER), is upregulated in UM associated with reduced survival rates. Knockdown of DERL1 by HSIEH et al. using siRNA reduced proliferation, migration, invasion, and metastasis in UM cells, increased its chemosensitivity to cisplatin, and diminished cancer stem cell properties [146]. The function of receptor tyrosine kinases (RTKs), which contribute to the epithelial‐mesenchymal transition (EMT) of tumors, is a considerable challenge in the treatment of UM. Recently, Wang et al. used RNAi technology to interfere with EMT caused by PDGFR‐α. In their research, they constructed a plasmid gene vector carrying shRNA against PDGFR‐α (PEI‐g‐PEG/PDGFR‐α shRNA) and transfected it to the OCM‐1 cell line. They observed reduced cell growth, cell migration, and angiogenesis. In vivo animal experiments demonstrated that PEI‐g‐PEG/PDGFR‐α shRNA effectively reduced the expression of PDGFR‐α and EMT markers; it also suppressed the growth of UM and decreased the development of blood vessel‐like structures [147]. Gene editing, achieved through the use of specific nucleases such as transcription activator‐like effector nucleases (TALENs), zinc‐finger nucleases (ZFNs), and CRISPR‐associated proteins (Cas), can also be employed in gene therapy. CRISPR‐Cas9 is particularly notable for its high efficiency and accuracy in making precise modifications to DNA sequences. Glinkina et al. used CRISPR in order to knockout IGF1R or PRKDC genes in uveal melanoma cell lines to assess whether depletion of these genes increases the anti‐tumor effects of everolimus, an mTOR inhibitor, in the treatment of UM. They observed that PRKDC‐knockout cells had altered morphology, and their growth was reduced significantly, likely because of cell cycle arrest [148]. CDS1 and CDS2 are highly sequence‐similar paralogs encoding vital enzymes contributing to the synthesis of phosphoinositides, which are involved in the MAPK, AKT, and other signaling [149]. In some cancers like uveal melanoma, downregulation of SCD1 causes the tumor cells to be vulnerable to CDS2 loss. A research study led by Chan et al. in 2025 employed CRISPR/Cas9 technology to deplete CDS2 and assessed its impact on the tumorigenic behaviors of UM cells in vivo. They reported a significant reduction in uveal melanoma cell growth in the xenografted mice [150].

Gene replacement is another mechanism of gene therapy, where the correct gene is introduced to the cells to replace the impaired causing a disease‐causing gene [151]. ncRNAs play a significant role in the development of UM, and inhibiting oncogenic types of them or inducing tumor‐suppressor ncRNAs could also be an effective approach. For example, miR‐30a‐5p is an anti‐tumor miRNA with a negative correlation of its expression with survival rates in patients. Very recently, Jiang et al. designed a small, easily synthesizable, biosafe, and stable construct, BiRDS, carrying miR‐30‐5p to UM cells. Their findings showed that induction of miR‐30‐5p using BiRDS into UM cells was followed by suppressed proliferation, migration, and invasion of UM cells while stimulating apoptosis. The in vivo analysis showed that BiRDS nanoeyedrops successfully penetrated the intricate ocular barrier and reached the fundus, resulting in the inhibition of uveal melanoma growth in a xenograft model. MiR‐30‐5p exerts its anti‐tumor effects via targeting E2F7 and stimulating apoptosis [152]. Also, introducing miR‐17‐3p mimic to UM cells by Wu et al. inhibited its target gene, MDM2, expression, leading to upregulated transcriptional activity of p53, followed by inhibiting UM cell proliferation, migration, and invasion, while stimulating cell apoptosis [153]. Although no direct gene replacement, introducing a wildtype version of an allele to UM cells harboring a mutation, has been reported in studies related to UM, there are studies in retinal genetic disorders that have successfully delivered coding sequences via AAVs to restore protein function [154], suggesting that a similar approach could be designed for UM treatment.

Another mechanism of gene therapy is suicide gene therapy, in which tumor cells are engineered to express an enzyme (e.g., HSV‐TK) that converts an inert prodrug into a cytotoxic agent [155]. Liu et al. designed a lentiviral vector encoding the cytosine deaminase (CD) gene, either alone or fused with the herpes simplex virus VP22 protein, to enhance intercellular distribution of the therapeutic enzyme. When the modified uveal melanoma OCM‐1 cells were treated with the prodrug 5‐fluorocytosine (5‐FC), intracellular CD converted it into the cytotoxic compound 5‐fluorouracil (5‐FU), effectively inducing apoptosis in tumor cells. In vitro assays showed significantly reduced proliferation and increased apoptosis in cells expressing VP22‐CD compared with CD alone. In a murine xenograft model, MRI and histological analyses indicated that tumor growth was inhibited following intratumoral delivery of the lentivirus followed by systemic 5‐FC administration, confirming extensive necrosis and decreased tumor volume. These findings demonstrate that VP22‐CD/5‐FC suicide gene therapy can exert potent antitumor activity in UM, highlighting its potential as a complementary or localized treatment option in future gene‐based therapies [156].

In recent years, there has been significant progress in the fields of stem cell technology [157]. In UV, the upregulation of the DNA damage response (DDR) could be linked to the activation of the PI3K/AKT pathway, contributing to treatment resistance. Zhang et al. demonstrated that the embryonic stem cell microenvironment (ESCMe) is able to repress malignancy in C918 cell lines by inhibiting proliferation, invasion, and tumorigenicity, with downregulation of the PI3K signaling pathway; moreover, ESCs engineered with a suicide gene (thymidine kinase) combined with ganciclovir treatment prevented ESC‐mediated teratoma formation in vivo [158]. Also, Liu et al. provided a valuable experimental model demonstrating the therapeutic relevance of stem‐cell‐based approaches in this malignancy. They co‐cultured C918 and normal cells with ESCs and found that ESCMe suppresses the proliferation, invasiveness, and tumorigenicity of C918 cells, while enhancing the proliferation of normal somatic cells both in vitro and in vivo. So, they suggested ESC transplantation as a safe and valuable strategy for treating UM [15]. However, the studies evaluating the employment of stem cell therapy for UM are restricted, and more investigations are required to validate their potential in the treatment of UM.

Immunomodulatory or oncolytic gene therapy combines the capacity to kill tumor cells directly via virus‐mediated lysis with the ability to elicit systemic anti‐tumor immune responses. Oncolytic viruses can be manipulated to selectively infect malignant cells and replicate in them. This causes the release of tumor antigens and danger signals, leading to stimulation of immune effectors such as dendritic cells, NK cells, and T cells. Inducing immunostimulatory transgenes or deletion of virulence genes further refines tumor specificity and safety. During a Phase 1 clinical trial, oncolytic adenovirus ICOVIR‐5 was administered intravenously in patients with cutaneous and uveal melanoma. In this trial, ICOVIR‐5 was found to exert an anti‐tumor effect in some uveal melanoma patients, accompanied by reduced tumor growth with low side effects. These findings support the feasibility of oncolytic gene therapy in uveal melanoma, while indicating that optimization in dose, administration route, and patient selection remains necessary [159].

**FIGURE 2:**
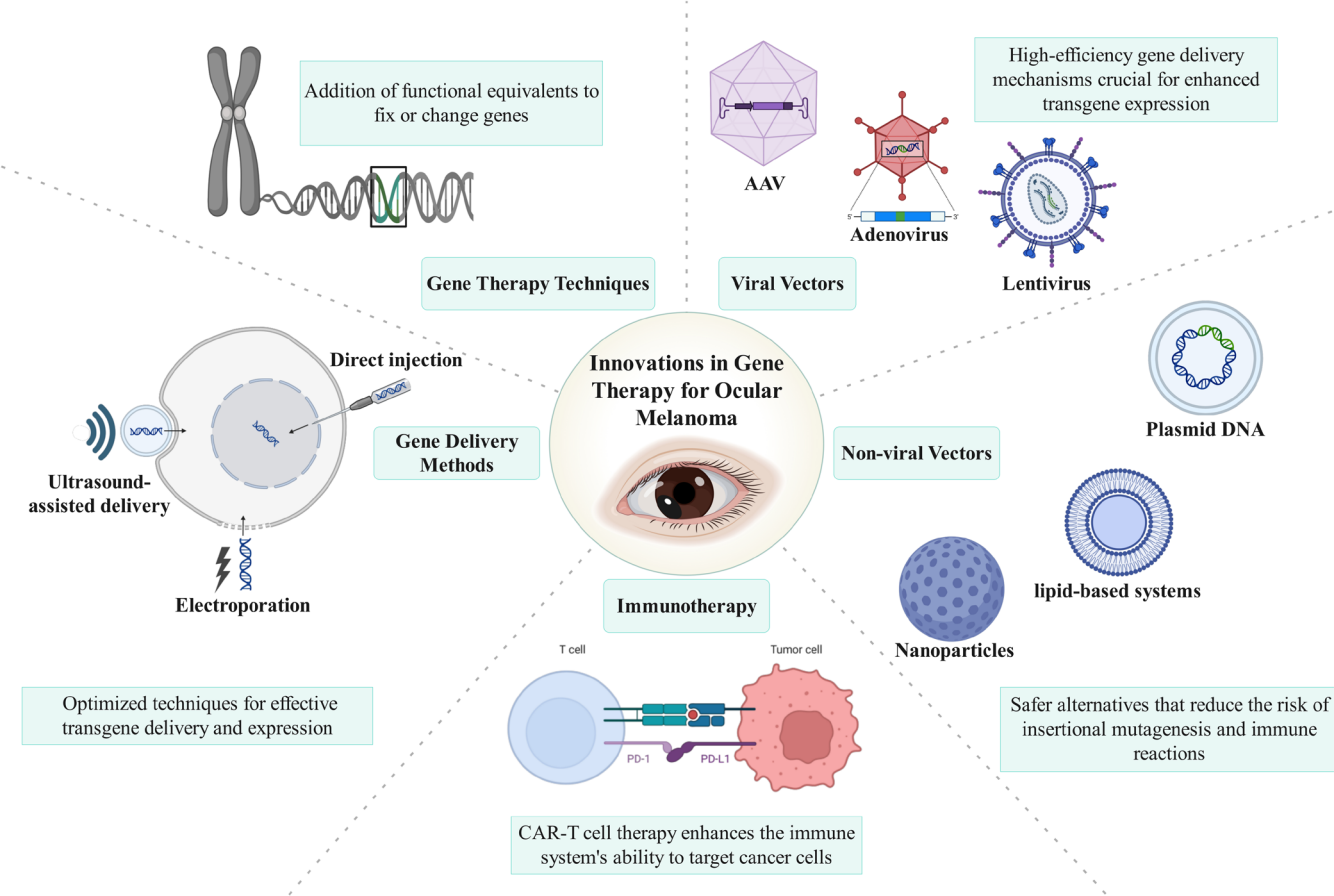
Schematic representation of gene delivery strategies used in ocular gene therapy. Both viral vectors (such as lentivirus, adenovirus, and AAV) and non‐viral methods (including liposomes and nanoparticles) are utilized for targeted gene transfer. Additionally, physical approaches such as electroporation and microinjection can facilitate gene delivery by transiently increasing cell membrane permeability. Each method offers unique advantages and limitations in terms of efficiency, safety, and tissue specificity.

Combining data on the GEP (Gene Expression Profile) and DNA mutations in ocular melanoma tumors could improve treatment outcomes by making it easier to find the most effective and personalized treatment plans [160]. The utilization of gene therapy has arisen as a potentially advantageous strategy for addressing ocular melanoma, an infrequent and highly aggressive form of eye cancer. There are numerous gene therapy modalities, each characterized by distinct mechanisms and potential advantages (Figure 2).

Due to its small size, distinct organization, and well‐separated structures, the eye serves as an excellent target for gene therapy. Additionally, many ocular disorders are linked to specific individual genes, which has facilitated more precise identification and understanding of these conditions. However, it is important to note that some eye disorders result from the combined influence of multiple genes working together. In December 2017, the FDA approved voretigene neparvovec‐rzyl (Luxturna), a gene therapy‐based drug, for the treatment of Leber congenital amaurosis (LCA) caused by biallelic retinal pigment epithelium‐specific 65‐kDa protein gene (RPE65). This was the first FDA‐approved gene therapy for an inherited retinal disease [161, 162]. The inability to control the lifespan of gene therapy and its irreversibility is one of the things that challenge the use of gene therapy. In addition to the different immune and inflammatory responses of different types of carriers and their subgroups, different methods of delivery, whether intravitreal, subretinal, or suprachoroidal, can disrupt the therapeutic effect or prevent repeated treatment on the patient. For example, in some cases, eye gene therapy causes inflammation and vision loss [163, 164, 165]. The first vector used for ocular gene therapy was adenovirus, which caused the death of a patient with systemic fever and liver damage in a patient with metabolic disease due to a strong immune response that caused inflammation and elimination of the transduced cells in a clinical study; currently, it is only used in the clinical gene therapy study of retinoblastoma [163, 166].

In three clinical trials for LCA patients with mutations in RPE65, AAV2 vectors carried a copy of RPE65, which had partial recovery results [164]. Lentiviruses are more capable of carrying transmissible genes than AAV, and by removing genes of the vector itself, it is possible to reduce the inflammatory response [167]. Non‐viral vectors for the transfer of large DNA‐like plasmids and oligodeoxynucleotides and RNA are created with the help of chemical and physical methods for the penetration of nanocomplexes into the cell membrane and inside the cell, and unlike viral vectors, there is less possibility.

The advantages of non‐viral vectors include a lack of immune response, mutagenesis, and insertion, high transfer capacity, and ease of production. However, the short‐term expression of the transferred genetic material is one of their disadvantages [163, 168]. Proliferation of uveal melanoma cells is reduced by the oncolytic virus Rigvir in MP41, 92‐1, and Mel‐202 cell lines in patients with UM, although more studies should be done by clinical trial [169]. The longer survival of patients with UM compared to cutaneous melanoma was observed in a very small amount and in a small number of patients, but these results can be in favor of ICOVIR5 in uveal melanoma disease compared to metastatic cutaneous melanoma [159]. Adenovirus H101, together with an alkylating agent such as DTIC in the treatment of UM in vivo, enhanced the effect of each other, which was shown in comparison with other groups [170]. An adenovirus engineered with the human mucin 1 antibody gene and the CD3 receptor—Ad5/3‐E2F‐d24‐aMUC1aCD3 (TILT‐321) replicates only in cells and produces large amounts of aMUC1aCD3 molecules in the tumor microenvironment, which causes tumor cell lysis [171, 172]. The results of using two groups of HSV1 virus and HSV equipped with GM‐CSF as the most effective anti‐tumor treatment on BALB/c‐nude mice showed that the HSV1 virus reduced tumor volume, and the virus HSV GMC‐CSF also showed antitumor effects [173]. Tumor volume reduction and long‐term survival in in vivo trials of HSV‐1 carrying cytosine deaminase *E.coli* in a study for melanoma yielded promising results for treatment [174]. The AAV5‐CMV‐Cre vector was obtained based on the UMass Vector Core and was injected with a glass needle and used to study the role of genes in the development and inhibition of genes in UV melanoma [175]. Reduction of tumor volume was observed in the study of suicide gene therapy lentiviral‐mediated CD/5‐FC suicide gene with the OCM‐1 cell line, and VP22 increased the cytotoxic effect of the CD gene [156, 176]. From the challenges of gene therapy in choroideremia, there is a lack of similar animal models to the functional manifestations and morphology of the disease and uncertainty of which retinal layer is affected most [163]. The results of gene therapy for choroideremia have been less successful than RPE65‐LCA, and all of them used the AAV vector [177]. In the first Phase 1 clinical study of human gene therapy in choroidoma in 2014, AAV2‐REP1 (10 genome particles) was injected under the fovea in one eye of six patients, and the follow‐up after the injection showed recovery during 3.5 years [178, 179, 180, 181].

Studies also showed that the amount of adenovirus infiltrating into the ocular tissues varies across the different portions of the eye. A minimally invasive way to inject into the ocular tissue is a subconjunctival injection using adenovirus, which leads to short‐term ocular transgene expression without causing hepatic injury and immune activation [182]. The gene expression inside AAV that was done through subconjunctival injection was not maintained for more than 112 days [183].

## 9 Gene Delivery Methods

Secure and effective gene delivery is the cornerstone and critical factor in successful gene therapy. Researchers have proposed several attributes for ideal gene delivery systems to ensure optimal gene therapy outcomes [184]. These systems should possess a wide‐ranging ability to incorporate genes at high transfection rates. They should also be very selective for targeting certain types of tumor cells, help genes stay expressed over time, and be easy to get and safe to administer. Gene delivery systems can be broadly classified into two primary categories: viral vectors and non‐viral vectors [185].

## 10 Viral‐Based Gene Delivery System

The first vectors to undergo extensive study and implementation were viral‐based gene delivery systems. These systems continue to be the go‐to choice for gene delivery [186]. Viral vectors work by taking advantage of the natural processes of viral infection and replication. This allows them to transport customized genetic material into specific host cells. However, to make these viral vectors more efficient, safer, and better at entering cells, genetic modifications are necessary [187].

Researchers have employed numerous viral vectors for gene delivery, including the adeno‐associated virus, lentivirus, adenovirus, retrovirus, and herpes simplex virus, among others. There have been a lot of research studies and experiments that have shown that viral vectors are better at transduction than non‐viral delivery systems. As an example, Verlengia et al. [188] used a modified herpes simplex virus vector to achieve transgene expression that lasted a long time. This shows that these vectors can be used in situations where transgene expression needs to last a long time. Additionally, viral vectors have some problems and restrictions, such as the ability to cause immune reactions, being toxic or mutagenic, being more expensive, and having limited cargo capacity in some virus types [187].

The adenovirus is a viral pathogen characterized by its DNA and is capable of effectively transducing both proliferating and nondividing cells. Utilizing adenovirus as a means of ocular gene therapy has proven to be highly effective in addressing transient changes in gene expression [189, 190]. Regrettably, Adenoviral Vectors (Ad) may still possess the capacity to elicit both adaptive and innate immune responses, resulting in undesirable outcomes such as cytotoxic T‐cell activation [191]. Contemporary iterations of Ad have undergone genetic manipulation by removing their complete genome while retaining inverted terminal repeats and encapsulating genes. This process aims to minimize immunological reactions and create more capacity for accommodating more extensive medicinal content [191, 192]. One concern that arises from widespread viral genome removal is the potential reactivation of immunological responses in infected cells. Innate immune receptors, which recognize viruses and trigger an immune response based on conserved molecular patterns, are responsible for this reactivation [193]. However, the use of recombinant Ad (rAd) vectors has proven to be effective in ocular gene therapy.

For ocular gene therapy trials, the most widely utilized vector is the adeno‐associated virus (AAV). The Parvoviridae family classifies AAVs as a non‐enveloped virus [194]. It is characterized by its small size, measuring approximately 25 nm, and its replication‐defective nature. AAVs possess a single‐stranded DNA genome. The gene therapy vector has undergone evaluation for several diseases, including metabolic, hematological, ophthalmological, muscular, and infectious conditions [189].

AAVs can deliver genetic material in the form of an extragenomic circular episome, which means they can do this without adding it to the human genome. This method is used to significantly reduce the likelihood of insertional oncogenesis. In a manner akin to adenovirus, AAVs possess the capability to infect both actively proliferating and quiescent cells [195, 196]. The restricted carrying capacity of AAVs poses a constraint on their utility in gene transfer for some disorders, such as Usher syndrome, which includes large genes [197]. Unlike adenoviruses, AAVs cause less severe innate and adaptive immune responses, which makes it easier to establish transgene expression that lasts. The aforementioned attributes render AAV vectors very suitable for implementation in a diverse range of chronic ocular disorders [198, 199].

Due to its complex structure and single‐stranded RNA composition, researchers have extensively investigated the lentivirus as a vector. Like the first two vectors, it can cause stable transduction in both dividing and nondividing cells in a wide range of target organs [167].

A lentivirus made from the equine infectious anemia virus (EIAV) has been used in clinical settings to deliver transgenes that stop corneal neovascularization by adding endostatin and angiostatin genes. Using lentiviral vectors to deliver the interleukin‐10 gene led to a significant rise in the survival rate of corneal transplants [200]. Furthermore, Smad7 gene expression significantly decreased subepithelial fibrosis in rats' corneal stroma. The lentiviral delivery of therapeutic genes resulted in a decrease in TGF/Smad signaling due to lower Smad2 phosphorylation. Furthermore, Smad7 downregulated the expression of pro‐fibrotic TGF‐2 [201]. Although lentiviruses are powerful gene delivery vectors, they have some disadvantages, including the risk of insertional mutagenesis and the potential for acting as tumor promoters via activation of oncogenes or inhibition of tumor suppressor genes. Furthermore, they can have restrictions in terms of stable expression and may produce lower titers than other viral vectors [202].

## 11 Non‐Viral‐Based Gene Delivery System

In the past few decades, there has been remarkable progress in the advancement of diverse non‐viral gene delivery techniques. These methods have undergone extensive testing to ensure their safety and effectiveness in delivering genetic material. As a result, there has been a growing interest in these techniques as a means to overcome the constraints associated with viral vectors. Li et al. [203] proposed the categorization of non‐viral gene delivery methods. They fall into three main categories: physically mediated approaches, chemical vectors, and biological methods.

Researchers have explored numerous physical mechanism‐based gene delivery techniques, including microinjection, ultrasound‐assisted microbubble delivery, microparticle bombardment, and electroporation [204]. These techniques have gained substantial attention from researchers over recent years due to their safe and effective gene delivery capabilities, in contrast to viral vectors. Within the realm of chemical vectors, two primary categories have gained prominence: cationic polymers and cationic liposomes [205]. However, challenges such as limited transfection efficiency and toxicity hinder their broader adoption [206, 207]. To address these issues, alternative options have emerged, such as shell nanoparticles and polymeric nanoparticles, which serve as substitutes for liposomes [208]. Non‐viral biological vectors encompass both bacteria and specific mammalian cells. Different types of bacteria have been used as gene carriers by researchers. These include weakened forms of Bifidobacteria, Salmonella, Shigella, Clostridia, Yersinia, Listeria, and nonpathogenic *Escherichia coli* [209]. Among mammalian cells, mesenchymal stem cells and hematological cells stand out as prominent choices for gene therapy vectors [210, 211]. Non‐viral vectors offer a range of advantages over viral vectors. These include enhanced safety profiles for various non‐viral methods, such as physically mediated delivery. Another notable advantage is the capacity to transfer larger genes, a feat unattainable with viral vectors. However, several limitations have been identified related to non‐viral vectors; for example, unmodified siRNA can be subject to degradation by serum endonucleases and can activate innate immunity upon intravenous administration. These encompass notably lower transfection efficiency and suboptimal transgene expression [212].

Researchers are studying options for ocular gene delivery without viral vectors. Non‐viral vectors, like naked plasmid DNA, oligonucleotides, and RNA, are less likely to cause insertional mutagenesis and immune responses. They can carry more information, be given more than once, and be easier to make in large quantities. Unless complexed with other chemical molecules or physically forced into cells for nuclear import, naked DNA molecules cannot transduce cells [213].

In recent studies, the field of DNA nanoformulations has attracted significant attention and has been the subject of comprehensive reviews. Therefore, we won't delve into a detailed discussion of this topic here. Lipid‐based delivery systems [214, 215], polymers [216], physical particles [217], and functionalized cell‐penetrating peptides are the chemical delivery methods that have been studied the most. Although animal models have demonstrated retinal cell transduction and therapeutic effects, human trials for ocular gene therapy have not evaluated DNA nanoparticles (NPs). This is primarily due to the limitations of low and/or short‐term transduction [218].

Physical methods exhibit greater variability and employ many techniques to facilitate the transfer of genetic information into cells. Various examples of techniques utilized in scientific research and medical applications include electrical pulses, ultrasound, magnetic fields, gene guns, and lasers [218, 219]. Plasmid DNA electrotransfection stands out as a particularly promising approach among the various methods under exploration for ocular gene delivery. Currently, a first clinical trial is underway, which holds enormous potential for the development of novel treatment strategies for sight‐threatening disorders [218].

Indeed, gene therapy approaches are still being investigated in experimental contexts and clinical trials. Regarding the current standard of care for UM, gene therapy is not now used as a first‐line treatment method for treating UM patients. Radiotherapy (such as plaque brachytherapy), surgical resection, and enucleation, depending on tumor size and location, are employed for the management of this disease, and gene therapy is unlikely to replace them. However, it may hold significant promise to localize in the second or third line approaches, particularly in patients with metastatic or treatment‐resistant diseases, in situations where conventional therapies do not work. In this condition, gene therapy may be employed as an adjunct treatment to improve patients' outcomes.

## 12 Challenges of the Application of Gene Therapy for Uveal Melanoma

Possibly, this now raises an important question for readers: given the compelling need for gene therapy‐based treatments for UM and the encouraging findings reported from studies investigating this issue, why have so few gene therapy drugs been approved for this disease? And why has gene therapy not yet been able to replace more conventional therapeutic approaches?

Despite the exciting prospects that gene therapy offers for targeted cancer treatment, several challenges have hindered the successful application of gene therapy in UM. Although preclinical data often suggest promising results, translating these findings into effective and safe therapies remains a complex project. A major barrier is the effective delivery of therapeutic genes specifically to uveal melanoma cells. As we described previously, the selection of an appropriate vector targeting the ocular melanoma cells without off‐target effects, with the capability of carrying the target gene with a specific size, and with a high delivery efficiency is challenging. Non‐viral vectors have low delivery efficiency, while viral vectors lack this problem, but they may have oncogenic and immune‐stimulatory effects [220, 221].

A further complication arises from the genetic heterogeneity of UM tumors. Since not all patients carry the identical mutations like GNAQ, GNA11, and BAP1, a gene therapy targeting one mutation may not be applicable to all. This variability necessitates a more personalized approach, which complicates trial design related to every mutation [58].

In addition, short‐lived gene expression is a limitation, especially with non‐integrating vectors or non‐viral delivery systems such as liposomes or nanoparticles. While these systems offer improved safety, they often fail to maintain therapeutic gene activity over time, requiring repeat dosing strategies [222, 223].

Also, ethical and long‐term safety concerns remain unresolved. Gene therapy is accompanied by the risk of insertional mutagenesis, especially when integrating vectors are used, which could activate oncogenes or disrupt tumor suppressor genes. These risks necessitate long‐term follow‐up, adding further complexity to trial planning [224].

Generally, it is possible that the immune system of the body reacts to the vector carrying the target gene, preventing it from effectively working and treating the disease. However, it is reported that the immunologic characteristics of the eyes are exceptional and allow the gene therapy to progress [16].

Moreover, clinical trials investigating the gene therapy effects on UM are limited, and most gene therapy research in UM is still in the preclinical phase, and the speed of translating preclinical studies to clinical application is slow due to strict safety evaluations and limited patient recruitment.

## 13 Conclusion and Future Perspective

Gene therapy offers a viable treatment approach for ocular melanoma, a rare and severe kind of eye cancer. Scientists have examined many methods of gene therapy, each with its own unique processes and benefits. Adenoviruses and AAVs are important tools for delivering genes in ocular melanoma gene therapy. Adenoviruses are capable of efficiently expressing transgenes at a high level, but only for a brief duration, making them advantageous for cancer treatment. On the other hand, AAVs can sustain transgene expression for an extended period, making them ideal for managing chronic illnesses. Animal models with iris melanoma have been treated with adenovirus‐mediated gene transfer in preclinical studies, showing that it is possible and works. However, further research involving larger sample sizes and longer follow‐up is necessary to evaluate the safety and efficacy of this approach in treating ocular melanoma. Gene therapy has potential as a treatment option for conjunctival melanoma (CJM), a rare and difficult kind of ocular melanoma, because it shares features with other melanomas that have responded well to gene therapy. Nevertheless, at this time, there have been no trials conducted, especially to assess the effectiveness of gene therapy for CJM. Putting together information about gene expression patterns and DNA changes in ocular melanoma tumors could improve treatment outcomes by creating more personalized and effective gene therapy plans. Further research is needed to determine the feasibility and efficacy of gene therapy for CJM. Overall, gene therapy is facing several limitations and challenges in the treatment of UM. But it is promising that future studies will overcome these challenges. One of the solutions to remove obstacles of gene therapy is the engineering of precision vectors designed to balance targeting specificity and safety. For instance, AAV vectors with tumor‐selective promoters or microRNA‐responsive cassettes may reduce off‐target expression while preserving high transduction efficiency. A combination of viral–nonviral systems, or cell‐assisted viral delivery, such as the delivery of oncolytic viruses by MSCs, also may be promising solutions to improve tumor penetration and reduce immunogenicity. By designing vectors that are less immunogenic and more specific to tumor cells, identifying and targeting specific mutations or pathways involved in UM development, and developing new approaches for delivering target genes to the uvea, such as local delivery or cell‐specific targeting. Understanding the genetic characteristics of ocular melanoma allows researchers to develop targeted therapies that may lead to better outcomes for patients with this rare form of cancer. To confront genetic heterogeneity in UM, future strategies should gravitate toward precision, mutation‐guided therapies. Integration of comprehensive tumor sequencing and CRISPR‐based custom editing allows tailoring interventions to GNAQ, GNA11, CYSLTR2, or BAP1 status per patient. Moreover, combining gene therapy with immunotherapies or small‐molecule inhibitors may control subclonal populations resistant to monotherapies. As described, the risk of tumorigenesis by insertional mutagenesis is also inevitable. To reduce the risks associated with this issue, base editing provides a precise, non‐integrating method for gene correction, thereby preventing insertional mutagenesis. Additionally, designing vectors with inducible suicide genes allows clinicians to terminate transgene expression in case of adverse events. In parallel, establishing long‐term patient follow‐up registries is essential for monitoring the safety and outcomes of ocular gene therapy.

Innovative technologies like sustained‐release systems, including ocular hydrogels and biodegradable nanoparticles, may be logical solutions to extend gene expression duration in the eye, thereby decreasing the need for repetitive administrations. Even though an immune reaction is a major barrier to gene therapy, the eye represents an immune‐privileged site, providing a unique opportunity for localized gene delivery. In the future, targeted administration approaches, such as intravitreal or suprachoroidal injections, may be used alongside immunologically inert or immune‐modulatory vectors to optimize ocular tolerance.

Overall, the future perspective suggests that by combining accurate vector engineering, individualized genomic approaches, regulated expression systems, and enhanced biosafety protocols, gene therapy has the potential to develop from a preliminary idea into a reliable and long‐lasting clinical treatment for uveal melanoma. Additionally, exploring alternative treatment options alongside gene therapy may provide a more comprehensive approach to managing this challenging disease.

## Author Contributions

A.Z. and V.G. contributed to the conceptualization, investigation, and writing of the original draft. Z.M., M.S., and A.B. contributed to the writing of the original draft. S.T., H.G., and M.S. contributed to writing, review and editing. H.F., M.S.S.M., and S.P. prepared the figures and tables. A.S., F.A.B., and Q.B. contributed to conceptualization, supervision, project administration, and verifying the manuscript before submission.

## Funding

The authors have nothing to report.

## Ethics Statement

The authors have nothing to report.

## Conflicts of Interest

The authors declare no conflicts of interest.

## Data Availability

Data sharing not applicable to this article as no datasets were generated or analyzed during the current study.
